# Supplementary material for: Cytoplasmic expression of C-MYC protein is associated with risk stratification of mantle cell lymphoma
Source: PeerJ. 2017 Jun 13;5:e3457. doi: 10.7717/peerj.3457 (PMC5472035; doi:10.7717/peerj.3457)
Supplement: Supplemental Information 2 [file peerj-05-3457-s002.docx]

| Variable | n |
| --- | --- |
| ***Sex*** | 64 |
| Male | 51 (79.7%) |
| Female | 13 (20.3%) |
| ***Age (years)*** | 60.05±10.80 |
| ***WBC (10^9^/L)*** | 7.77±3.26 |
| ***LDH (U/L)*** | 333.39±232.91 |
| ***ECOG performance status*** | 64 |
| **0** | 46 (71.9%) |
| **1** | 14 (21.9%) |
| **2** | 4 (6.2%) |
| ***Ann Arbor stage*** | 64 |
| Ⅱ | 3 (4.69%) |
| Ⅲ | 32 (50.0%) |
| Ⅳ | 29 (45.31%) |
| ***Location*** | 64 |
| Nodal | 46 (71.9%) |
| Extra-nodal | 18 (28.1%) |
| ***B symptoms*** | 64 |
| No | 29 (45.3%) |
| Yes | 35 (54.7%) |
| ***Risk group*** | 31 |
| low | 11 (35.5%) |
| intermediate | 16 (51.6%) |
| high | 4 (12.9%) |
| ***Treatment*** | 31 |
| CHOP | 13 (41.9%) |
| CHOPE | 3 (9.7%) |
| GDP | 1 (3.2%) |
| Hyper-CVAD | 3 (9.7%) |
| RCHOP | 11 (35.4%) |
| ***Response*** | 31 |
| CR | 5 (7.8%) |
| PR | 17 (26.56%) |
| PD | 9 (14.1%) |
| ***Cytoplasmic C-MYC*** | 64 |
| - | 19 (29.7%) |
| + | 25 (39.1%) |
| ++ | 20 (31.2%) |
| ***Nuclear C- MYC (%)*** | 14±17 |
| ***P53(%)*** | 19±22 |
| ***CD8+TIL*** | 134.26±55.75 |
| ***PD-L1*** |  |
| - | 15 (23.4%) |
| + | 28 (43.8%) |
| ++ | 4 (6.3%) |
| +++ | 1 (1.5%) |
| ***SOX11(%)*** | 30±30 |
| ***Ki67(%)*** | 42±24 |
